# Elucidating the Venom Diversity in Sri Lankan Spectacled Cobra (*Naja naja*) through *De Novo* Venom Gland Transcriptomics, Venom Proteomics and Toxicity Neutralization

**DOI:** 10.3390/toxins13080558

**Published:** 2021-08-10

**Authors:** Kin Ying Wong, Kae Yi Tan, Nget Hong Tan, Christeine Ariaranee Gnanathasan, Choo Hock Tan

**Affiliations:** 1 Venom Research and Toxicology Laboratory, Department of Pharmacology, Faculty of Medicine, University of Malaya, Kuala Lumpur 50603, Malaysia; kinying12@gmail.com; 2 Protein and Interactomics Laboratory, Department of Molecular Medicine, Faculty of Medicine, University of Malaya, Kuala Lumpur 50603, Malaysia; tanngethong@yahoo.com.sg; 3 Department of Clinical Medicine, Faculty of Medicine, University of Colombo, Colombo 00800, Sri Lanka; ariaraneegnanathasan@gmail.com

**Keywords:** Indian cobra, venomics, venom-induced toxicity, antivenom, neutralization

## Abstract

Inadequate effectiveness of Indian antivenoms in treating envenomation caused by the Spectacled Cobra/Indian Cobra (*Naja naja*) in Sri Lanka has been attributed to geographical variations in the venom composition. This study investigated the *de novo* venom-gland transcriptomics and venom proteomics of the Sri Lankan *N. naja* (NN-SL) to elucidate its toxin gene diversity and venom variability. The neutralization efficacy of a commonly used Indian antivenom product in Sri Lanka was examined against the lethality induced by NN-SL venom in mice. The transcriptomic study revealed high expression of 22 toxin genes families in NN-SL, constituting 46.55% of total transcript abundance. Three-finger toxins (3FTX) were the most diversely and abundantly expressed (87.54% of toxin gene expression), consistent with the dominance of 3FTX in the venom proteome (72.19% of total venom proteins). The 3FTX were predominantly S-type cytotoxins/cardiotoxins (CTX) and α-neurotoxins of long-chain or short-chain subtypes (α-NTX). CTX and α-NTX are implicated in local tissue necrosis and fatal neuromuscular paralysis, respectively, in envenomation caused by NN-SL. Intra-species variations in the toxin gene sequences and expression levels were apparent between NN-SL and other geographical specimens of *N. naja*, suggesting potential antigenic diversity that impacts antivenom effectiveness. This was demonstrated by limited potency (0.74 mg venom/ml antivenom) of the Indian polyvalent antivenom (VPAV) in neutralizing the NN-SL venom. A pan-regional antivenom with improved efficacy to treat *N. naja* envenomation is needed.

## 1. Introduction

Worldwide, it is estimated that 1.8–2.7 million cases of snakebite envenomation occur each year, with fatalities ranging from around 81,000 to 138,000, and around three times as many continue to suffer permanent complications resulting from amputations, deformity and chronic organ failures [1]. Snakebite envenomation is an important cause of morbidity and mortality that heavily affects impoverished populations in South Asia and Southeast Asia, where the highest incidence of venomous snakebites in the world has been reported [2,3]. The sociodemographic and agriculture-related occupational profile of the population contributes to the increased risk of snake–human encounters in the region, which is a biodiversity hotspot of venomous snakes [4,5].

In South Asia, the major venomous snake species responsible for causing snakebite envenomation are typically referred to as the “Big Four”, which include the spectacled cobra (*Naja naja*), common krait (*Bungarus caeruleus*), Russell’s viper (*Daboia russelii*) and saw-scaled viper (*Echis carinatus*). The diverse topology and socio-ecological systems in South Asia, however, greatly influence the biogeographical distribution of the snakes and their adaptation to different habitats. This would have modulated the venom phenotype in terms of toxin composition, thus contributing to intra-species variation in the snake venom [6,7,8,9,10,11]. Sri Lanka, which is a tropical island country in the Indian Ocean, is long separated by the Palk Strait and biogeographically distinct from the Indian Peninsula. This has an impact on the management of snakebite envenomation in South Asia, as intra-species venom variation arose from biogeographical factor greatly affects the efficacy of Indian antivenom product used in Sri Lanka [12,13]. Based on recent hospital records and community surveys, over 33,000 snakebite cases and 400 deaths occur in Sri Lanka annually [14]. The Spectacled Cobra (*N. naja*, also known as Indian Cobra, Binocellate Cobra), which is widely distributed in the island country, is a leading cause of snakebite envenomation, with toxicity manifested as systemic neuromuscular paralysis and local tissue damage [15,16]. As there are no domestic antivenoms produced in Sri Lanka, antivenoms clinically used in the country are imported Indian polyvalent antivenom products, which are raised against the Big Four commonly from a specific region (Tamil Nadu) in India. For decades, the use of Indian antivenom in Sri Lanka has been questioned for low efficacy in treating envenomation caused by the local snake species. Presumably, the discrepancy of antivenom efficacy is due to snake venom variability between Sri Lanka and India [7,12,17,18,19,20]. To warrant safe and effective antivenom use, a robust assessment of imported antivenoms is necessary, and in the long run, developing a domestic antivenom product specific for native venomous snakes in Sri Lanka is the way forward [21,22]. 

The knowledge of snake venom composition details is essential to elucidate the intra-species venom variability. Current -omics technologies allow high throughput sequencing and identification of snake venom proteins and toxin genes, thereby unveiling the diversity of toxin genes and the dynamic range of venom complexities in many species, including widely distributed venomous snakes such as the cobras. Previous enzymatic, proteomic and transcriptomic studies of *Naja* species have revealed significant inter- and intra-species variation in venom composition and toxin gene diversity [6,7,23,24,25,26,27,28,29,30]. More recently, the genome of Indian *N. naja* (Kerala State) was reported, and the venom-gland transcriptomes of six cobra specimens sourced from Kerala in India and Kentucky Reptile Zoo in USA were profiled [31]. The study identified 15 toxin genes in the Indian Cobra *N. naja*, with three-finger toxins (3FTX) being the most highly expressed. Striking variations were, however, noted in the diversity and expression of the toxin genes across the different specimens even though they were sourced from the same species of the Indian origin. Another recent proteomic study further showed substantial intraspecific venom variation among six *N. naja* specimens from various geographical locales in India, including the Deccan plateau, Gangetic plain, semi-arid, desert and coastal regions [6]. The variation in venom composition resulted in differential venom toxicity and disparity in the efficacy of antivenom neutralization. It was observed that Indian cobra venom samples with a higher abundance of neurotoxins appeared to be less effectively neutralized by existing Indian antivenom products in the market [6]. In light of the finding, the toxin gene profile and venom proteome of the Sri Lankan *N. naja* are anticipated to vary markedly from the Indian counterpart, but this has not been investigated in depth. Earlier, there was one proteomic study comparing venom proteomes between the Indian and Sri Lankan *N. naja*, in which merely six toxin families comprising 26 and 25 proteins, respectively, were identified [7]. Additionally, the study showed that Indian VINS polyvalent antivenom bound less avidly to the Sri Lankan cobra venom toxins than the homologous Indian cobra toxins, supporting that Indian antivenom was generally less effective in treating cobra bite envenomation in Sri Lanka [20]. 

To elucidate the toxin gene diversity of the Sri Lankan Spectacled Cobra (*N. naja*), this study investigated the *de novo* venom-gland transcriptome of the snake applying next-generation sequencing techniques, and the venom proteomics of the snake was studied through a protein decomplexation strategy [32]. The neutralization efficacy of a commonly used Indian antivenom product (VINS polyvalent antivenom) was also assessed against the lethality induced by the venom *in vivo*. Findings from the study shed light on the variation of snake venom toxins in *N. naja*, and provide insights into the optimization of snakebite management in Sri Lanka. 

## 2. Results and Discussion

### 2.1. De Novo RNA Sequencing and Transcriptome Assembly

*De novo* sequencing on Illumina platforms generated 54,477,234 paired-end raw reads from the venom gland tissue of the Sri Lankan *Naja naja* (NN-SL). After adapter trimming and quality filtering, a total of 51,595,008 clean reads were obtained. The clean reads were assembled into 106,637 contigs (N50 = 361) by Trinity program and subsequently clustered into 53,595 Unigenes (N50 = 676) (Table 1). Low-frequency transcripts were filtered at FPKM (fragments per kilobase of transcript per million mapped reads) below 1, streamlining the number of transcripts to 50,922 for expressed genes in the venom gland. The genes were further categorized based on their functional annotations: (i) toxin (80 transcripts), with known or putative toxinological role; (ii) non-toxin (24,226 transcripts), with encoded cellular or housekeeping proteins; (iii) unidentified (26,616 transcripts), where the gene identities could not be determined (Supplementary File S1). Among these, the toxin group constituted the most abundantly expressed genes with the highest percentage of total FPKM (46.55% of total gene expression) and redundancy value (5699.14 FPKM/transcript), as compared to the non-toxins (FPKM = 34.33%, redundancy = 13.88 FPKM/transcript) and unidentified (FPKM = 19.12%, redundancy = 7.03 FPKM/transcript) groups. The dominant expression and high redundancy of toxin genes in the venom gland transcriptome of NN-SL were consistent with findings reported for various elapid snakes, including the *Hydrophis curtus* (48.18% of total FPKM) [33], *Ophiophagus hannah* (35.3%) [34], Malaysian and Thai *Naja kaouthia* (41.2% and 82%, respectively) [27], *Naja sumatrana* (79.2%) [35], Chinese *Naja atra* and *Bungarus multicinctus* (70% of expressed sequences tags) [36] and American *Micrurus fulvius* (45.8%) [37]. 

### 2.2. Venom Gland Transcriptome of NN-SL

**Figure 1:**
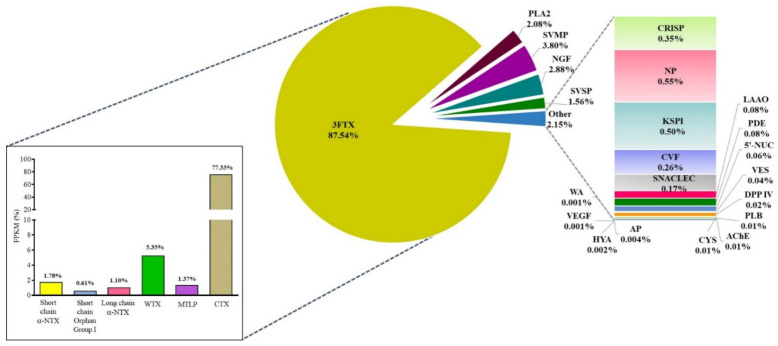
*De novo* venom-gland transcriptome of Sri Lankan *Naja naja*: the expression profile of toxin genes in the venom gland of spectacled cobra, *Naja naja* from Sri Lanka. Percentages indicate the relative abundances of transcripts by total toxin FPKM, sorted according to gene family/subfamily. Abbreviations: 3FTX, three-finger toxins; α-NTX, α-neurotoxin; WTX, weak neurotoxin; MTLP, muscarinic toxin-like protein; CTX, cytotoxin/cardiotoxin; PLA_2_, phospholipase A_2_; SVMP, snake venom metalloproteinases; NGF, nerve growth factor; SVSP, snake venom serine protease; CRISP, cysteine-rich secretory protein; NP, natriuretic peptide; KSPI, Kunitz-type serine protease inhibitor; CVF, cobra venom factor; SNACLEC, snake C-type lectins; LAAO, L-amino acid oxidase; PDE, phosphodiesterase; 5’-NUC, 5’-nucleotidase; VES, vespryn; DPP IV, dipeptidylpeptidase IV; PLB, phospholipase B; AChE, acetylcholinesterase; CYS, cystatin; AP, aminopeptidase; HYA, hyaluronidase; VEGF, vascular endothelial growth factor; WA, waprin. FPKM, fragments per kilobase of transcript per million mapped reads.

A total of 22 toxin gene families comprising 80 non-redundant transcripts were identified in the venom gland transcriptome of NN-SL (Figure 1). Of these, the three-finger toxins (3FTX) were the most abundantly and diversely expressed (87.54% of total toxin FPKM, 13 transcripts), followed by snake venom metalloproteinase (SVMP, 3.80%, 18 transcripts), nerve growth factor (NGF, 2.88%, 1 transcripts), phospholipase A_2_ (PLA_2_, 2.08%, 5 transcripts) and snake venom serine protease (SVSP, 1.56%, 3 transcripts) (Table 2). Other toxin gene families included acetylcholinesterase (AChE), aminopeptidase (AP), cysteine-rich secretory protein (CRISP), cobra venom factor (CVF), cystatin (CYS), dipeptidyl peptidase IV (DPP IV), hyaluronidase (HYA), L-amino acid oxidase (LAAO), natriuretic peptide (NP), Kunitz-type serine protease inhibitor (KSPI), snake C-type lectin (SNACLEC), phosphodiesterase (PDE), 5’-nucleotidase (5’-NUC), vespryn (VES), phospholipase B (PLB), vascular endothelial growth factor (VEGF) and waprin (WA), which were all minimally expressed (<1% of all toxin FPKM) and less diversified (1–4 transcripts each) (Table 2). All toxin transcripts from NN-SL were annotated based on sequence similarity with known toxins of elapid species, while approximately half of the toxin transcripts (mainly 3FTX) were matched to related cobra species (*N. atra*, *N. kaouthia*, *N. sputatrix*, *Naja mossambica*), implying conserved sequences of 3FTX genes in the genus. A total of 31 toxin transcripts were encoded in full-length sequences with >90% coverage of the gene sequences (Table 2).

### 2.3. Venom Proteome of NN-SL

**Figure 2:**
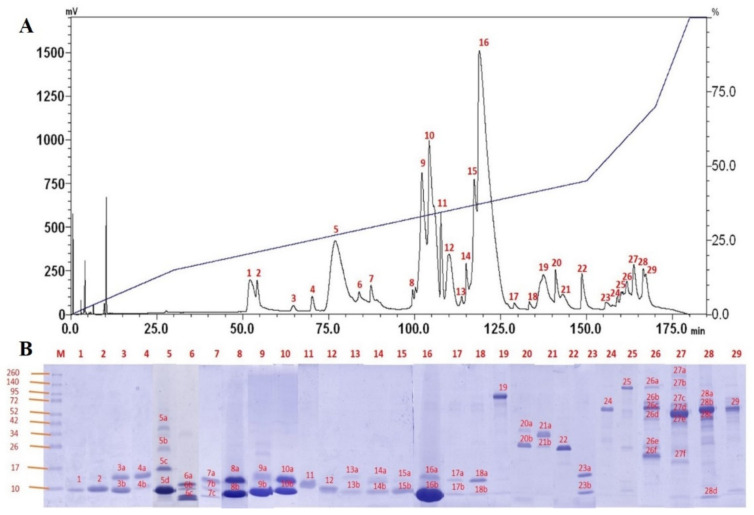
(**A**) C18 reverse-phase HPLC fractionation of Sri Lankan Spectacled Cobra (*Naja naja*) venom. (**B**) SDS-PAGE of the HPLC-fractionated venom proteins under 15% reducing conditions. M: Protein ladder with molecular weight 10–260 kDa.

Using C18 reverse-phase HPLC, the NN-SL venom was resolved into 29 protein fractions with increasing hydrophobicity (Figure 2A). The protein fractions were individually subjected to SDS-PAGE under 15% reducing conditions, and a total of 63 heterogeneous protein bands were yielded (Figure 2A,B). The decomplexation profile of NN-SL venom was comparable to that of several Asiatic *Naja* species reported, studied under similar experimental conditions [8,23,38,39]. In brief, the majority of NN-SL venom proteins were 7–20 kDa and eluted between 50 min and 135 min of reverse-phase chromatography (fractions 1–18), accounting for approximately 88% of total venom composition as estimated by the chromatogram peak areas. Proteins with moderate-to-high molecular weight (>25 kDa) were eluted in the subsequent 40 min and accounting for approximately 12% of total venom proteins.

**Figure 3:**
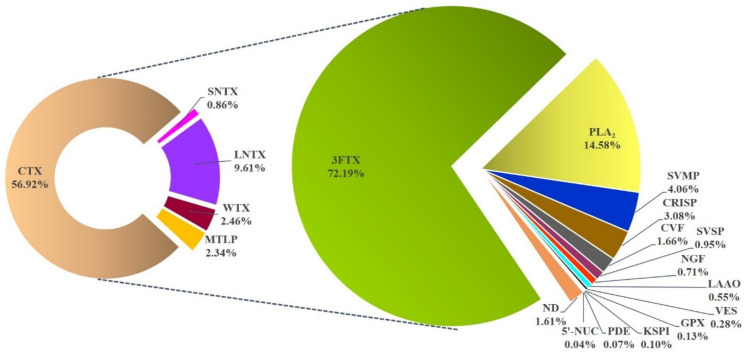
Venom proteome of the Sri Lankan Spectacled Cobra (*Naja naja*), profiled according to toxin families and relative protein abundances (% of total venom proteins). Abbreviations: 3FTX, three-finger toxins; SNTX, short neurotoxin; LNTX, long neurotoxin; WTX, weak neurotoxin; MTLP, muscarinic toxin; CTX, cytotoxin/cardiotoxin; PLA_2_, phospholipase A_2_; SVMP, snake venom metalloproteinase; CRISP, cysteine-rich secretory protein; CVF, cobra venom factor; SVSP, snake venom serine protease; NGF, nerve growth factor; LAAO, L-amino acid oxidase; VES, vespryn; GPX, glutathione peroxidase; KSPI, Kunitz-type serine protease inhibitor; PDE, phosphodiesterase; 5‘-NUC, 5’-nucleotidase; ND, not determined/ unidentified.

MALDI TOF/TOF and nano-ESI-LCMS/MS analyses of the in-gel digested peptides identified a total of 45 proteins that were clustered into 12 toxin families and a non-toxin protein family in the NN-SL venom proteome (Figure 3). The proteome was dominated by the low molecular weight 3FTX (72.19% of total venom proteins) and PLA_2_ (14.58%). The remaining ten toxin families collectively constituted less than 12% of total venom proteins, and these were snake venom metalloproteinase (SVMP, 4.06%), cysteine-rich secretory protein (CRISP, 3.08%), cobra venom factor (CVF, 1.66%), snake venom serine protease (SVSP, 0.95%), nerve growth factor (NGF, 0.71%), L-amino acid oxidase (LAAO, 0.55%), vespryn (VES, 0.28%), Kunitz-type serine protease inhibitor (KSPI, 0.10%), phosphodiesterase (PDE, 0.07%), 5’-nucleotidase (5’-NUC, 0.04%) and a non-toxin glutathione peroxidase (GPX, 0.13%) (Table 3). The dominating feature of 3FTX and PLA_2_ in NN-SL venom proteome follows a somewhat predictable trend as observed in virtually all previously reported venom proteomes of Asiatic cobras, notwithstanding variability in terms of the diversity (subtypes) and quantity (relative abundances) of their toxins [6,7,8,23,24,26,28,30,38,39,40,41,42,43,44]. 

Comparing between the venom gland transcriptome and venom proteome, we observed a significant correlation between the toxin gene expression (% of all toxin FPKM) and the venom protein abundance (% of total venom proteins) of NN-SL (Spearman correlation coefficient, r = 0.7062, *p* <0.05) (Supplementary Figure S1). Among the 22 toxin families identified in the venom gland transcriptome, 12 protein families (3FTX, PLA_2_, SVMP, NGF, SVSP, CRISP, KSPI, CVF, LAAO, PDE, VES, 5’-NUC) were detected at the proteomic level. The toxin genes found only at the transcriptomic level were primarily those with very low expression, including NP, SNACLEC, CYS, AChE, VEGF, DPP IV, PLB, AP, WA and HYA. These proteins might be present at an extremely low amount below the mass spectrometry detection limit, or, the translation of the mRNA had been negatively regulated post-transcriptionally [45]. 

The data of the mass spectrometry analysis, including peptide sequences, values of spectral mass/charge and relative abundances of proteins identified was provided in Supplementary File S2.

### 2.4. Three-Finger Toxins (3FTX)

**Figure 4:**
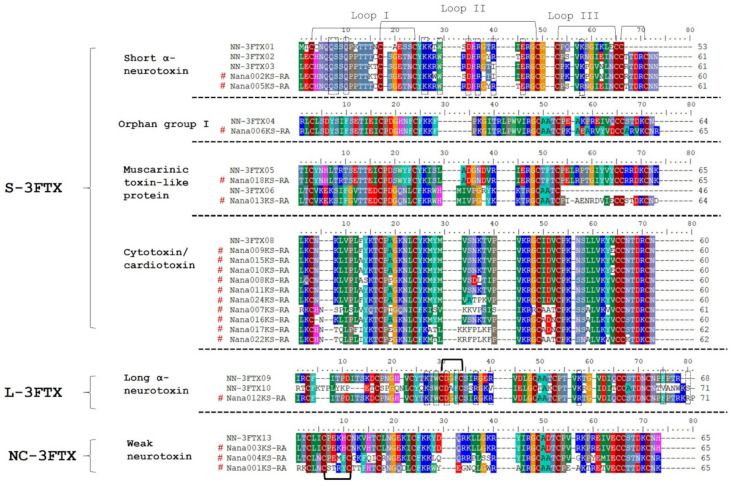
Multiple sequence alignment of the three-finger toxins (3FTX) transcripts from the venom gland transcriptome of *N. naja*. The alignment of 3FTX transcripts based on subfamilies: short-chain, long-chain and non-conventional toxins. The 3FTX transcripts obtained in the current study were aligned and compared with the gene sequence from the transcriptome of *N. naja* specimens sourced from Kerala, India and Kentucky Reptile Zoo, USA. # indicates the gene sequences of 3FTX retrieved from [31]. The black bracket indicates the disulfide bridges located at the first, second and third loop of 3FTX, respectively. The additional bracket denotes the fifth disulfide bridge located at the first and second loops of NC-3FTX and L-3FTX, respectively. Dotted square box in the amino acid sequence of short α-neurotoxins and long α-neurotoxins highlights the critical region of amino acids that bind to nAChR [66].

The high expression levels of 3FTX in the transcriptome and proteome of NN-SL support a pivotal role of these toxins in predation and pathogenesis of envenomation. Three-finger toxins are non-enzymatic polypeptides containing 60–74 amino acids with 8–10 conserved cysteinyl residues. The molecule adopts a three-finger-like structure that consists of three antiparallel ß-stranded loops extending from a small hydrophobic core cross-linked by conserved disulfide bridges [46,47]. Of the 13 diverse 3FTX genes expressed in NN-SL, eight belonged to the short-chain 3FTX (S-3FTX, characterized by the presence of four disulfide bonds); these were denoted as NN-3FTX01–08, comprising three short-chain alpha-neurotoxins (SNTX), one cytotoxin/cardiotoxin (CTX), three muscarinic toxin-like proteins (MTLP), and one orphan group I 3FTX (Table 2). The remaining five 3FTX genes were of long-chain 3FTX (L-3FTX, with 5 disulfide bonds), comprising four long-chain alpha-neurotoxins (LNTX) and one non-conventional 3FTX (NC-3FTX) (Table 2). The primary structures of the 3FTX were shown in Figure 4, illustrating the four conserved disulfide bonds between loop I and loop III of SC-3FTX, and the additional fifth disulfide bond present at the N-terminal loop I of NC-CTX and the central loop II of LNTX. 

Of the 13 3FTX genes identified in NN-SL, the CTX transcript NN-3FTX08 was the most abundantly expressed in its venom gland (77.33% of total toxin FPKM). The sequence of NN-3FTX08 was highly similar to Nana009KS-RA, the major CTX gene of *N. naja* from India with one amino acid substitution at the 52nd residue position (E^52^ → V^52^) [31] (Figure 4; note that the position in alignment figure is 64 due to artificial gapping). Both NN-3FTX08 and Nana009KS-RA are S-type CTX based on the presence of S^28^ residue (Figure 4, position in alignment figure: 35) in the protein, as proposed in the S-type/P-type CTX classification system [48,49]. The P-type CTX, as exemplified by CTX A3 from the Taiwanese Cobra, *N. atra*, has a more pronounced lipid-binding and membrane pore-forming activities than the S-type CTX [50,51], although the latter also exhibits cytotoxicity in human cell lines [52]. Between NN-3FTX08 and Nana009KS-RA, the substitution of polar, negatively charged glutamic acid (E^52^) in Nana009KS-RA to hydrophobic valine (V^52^) (Figure 4, position in alignment figure: 64) in NN-3FTX08 may result in increased hydrophobicity of loop III by strengthening the interaction of tyrosine (Y^51^, position in alignment figure: 63) with the lipid membrane [53], thereby causing a more pronounced lipolytic activity of the CTX. There is, however, a dearth of study that compares the clinical toxicity of *N. naja* envenomation between India and Sri Lanka to shed light on the potential difference in cytotoxicity of the venoms. Hence, studies with a focus on the comparative characterization of CTX of *N. naja* from different locales should be the subject of future research.

The relative protein abundance of CTX present in the NN-SL venom proteome was highly correlated with the CTX gene expression in the venom gland. The high abundance of CTX (56.92% of total venom proteins) is consistent with the development of severe tissue necrosis in envenomation caused by *N. naja* [13,20]. With a median lethal dose (LD_50_) of 1–2 ug/g in mice, cobra venom CTX usually do not cause a direct lethal activity [54,55] but are implicated in the local tissue damage and venom ophthalmia [56,57]. Cationic CTX are able to bind to the anionic phospholipid bilayer of cell membrane, thereby disrupting the membrane integrity, forming pore and initiating cell death [48,58,59]. CTX has also been known to elicit a cardiotoxic effect by causing systolic heart arrest in ex vivo experiments [60,61], albeit direct cardiotoxicity is rarely seen in cobra envenomation. From the evolutionary perspective, it has been suggested that the cytotoxic effect of CTX co-evolved with the hooding display behavior and aposematic coloration of cobras for defense purposes [62]. 

The alpha-neurotoxins are the principal lethal components (with LD_50_ of 0.05–0.2 mg/kg in mice) in cobra venoms [38,39,54,55,63]. These toxins are post-synaptic neurotoxins that bind to the nicotinic acetylcholine receptors (nAChR) at the neuromuscular junction, thereby interrupting neurotransmission, and rapidly resulting in descending paralysis as manifested clinically in cobra bite envenomation [64,65,66]. Three transcripts of short-chain α-neurotoxin (SNTX, NN-3FTX01-03) and four transcripts of long-chain α-neurotoxin (LNTX, NN-3FTX9-12) were identified in the venom gland transcriptome of NN-SL (Table 2). In comparison to alpha-neurotoxins of the Indian *N. naja*, the SNTX in NN-SL (NN-3FTX02) was found identical to Nana005KS-RA [31], whilst two amino acid substitutions occurred between NN-3FTX03 and Nana002KS-RA at loop II: W^27^ and S^29^ in Nana002KS-RA were substituted by R^27^ and R^29^ in NN-3FTX03, respectively, as shown in Figure 4 (positions in alignment figure: 28 and 34). The substitutions involving these polar amino acids (Trp, Ser and Arg) were predicted to be conservative based on the Sorting Intolerant from Tolerant (SIFT) algorithm [67,68], thus unlikely to result in a significant change of the SNTX activity. Meanwhile, NN-3FTX01 was not previously reported in the *N. naja* specimens sourced from India and Kentucky Reptile Zoo, hence it could be a diverging SNTX form unique to the Sri Lankan cobra population. 

The main transcript of LNTX in NN-SL (NN-3FTX09) was homologous to the LNTX transcript Nana012KS-RA of the Indian *N. naja*, with two amino acid substitutions that occurred at the residue positions of 35 and 49 (from NN-SL to the Indian Cobra of Kerala: E^35^ → K^35^ and K^49^ → R^49^) (Figure 4, positions in alignment figure: 39 and 58). The substitution of R^49^ with K^49^ (Arg and Lys are basic and charged) was conservative while the replacement involving E^35^ (acidic Glu) and K^35^ (basic Lys) might result in altered activity, although LNTX were typically the most lethal component in cobra venoms [54,55]. Irrespective of the subtle sequence variation between NN-SL and the Indian Cobra, these alpha-neurotoxins (SNTX and LNTX) bind to similar sites on the nicotinic acetylcholine receptors (nAChR) with high affinity [69]. The SNTX binds to the nAChR six- to seven-times faster and dissociates five- to nine-times faster than LNTX, which is known to cause relatively irreversible block compared to SNTX [70,71]. Toxin neutralization experiments, on the other hand, showed that most antivenoms neutralized SNTX less efficaciously compared to LNTX, implying that the SNTX has a lower immunogenicity than LNTX [54,55]. It should be noted though that the neutralization efficacy of antivenoms against the lethality of cobra venoms is generally low (potency <1 mg/mL in terms of the amount of venom completely neutralized by one mLof antivenom) [72,73], compared to the neutralization of viper and pit viper venoms in which the antivenom potency commonly exceeded 2 mg/mL [74,75,76]. The abundance of alpha-neurotoxins, therefore, appears to be the limiting factor of antivenom neutralization. Proteomic studies of cobra venoms should aim to characterize the quantitative toxin abundance in order to elucidate differences in the venom lethality and neutralization efficacy of antivenom [39]. 

The venom-gland transcriptomics also unveiled the genes of minor 3FTX including muscarinic toxin-like protein (MTLP), neurotoxin-like protein (Orphan Group I) and weak neurotoxin (WTX) (Table 2). These toxin genes, albeit lowly expressed, were rather conserved venom phenotype between NN-SL and *N. naja* specimens from different geographical locales [31] (Figure 4). The MTLP-encoding genes in NN-SL (NN-3FTX05 and NN-3FTX06) show sequences identical with those of the Indian Cobra (Nana018KS-RA and Nana013KS-RA). Similarly, the WTX genes in NN-SL (NN-3FTX13) and the Indian Cobra (Nana003KS-RA) were also identically matched by their sequences (Figure 4). The genes encoding Orphan Group I neurotoxin-like protein in NN-SL (NN-3FTX04) were similar to the Indian Cobra (Nana006KS-RA) except with some amino acid differences in loop III and C-terminal region (Figure 4). These 3FTX genes were expressed in low amount into venom proteome, accounting for ~2% of total venom proteins of NN-SL (Figure 3). These minor toxins have been shown to have low toxicity and lack lethal activity [77,78,79]. 

### 2.5. Phospholipase A_2_ (PLA_2_)

**Figure 5:**
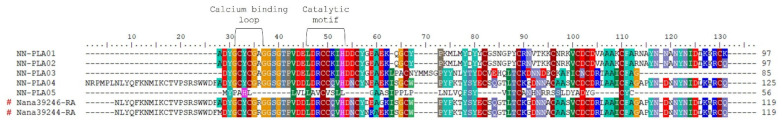
Comparison of PLA_2_ transcripts of NN-SL and *N. naja* specimens from Kerala, India and Kentucky Reptile Zoo, USA. # indicates the gene sequences of PLA_2_ retrieved from [31]. Top brackets indicate calcium-binding loop and catalytic motif.

A total of five PLA_2_ transcripts (NN-PLA01–05, Table 2) were identified in the venom-gland transcriptome of NN-SL. NN-PLA04 was the most abundantly expressed secretory PLA_2_ transcript, with a sequence highly similar (>90%) to the PLA_2_ transcripts (Nana39246-RA and Nana39244-RA) from the Indian Cobra of Kerala [31] (Figure 5). NN-PLA04 belongs to the snake venom PLA_2_ of Group IA family, and its calculated pI (5.22) indicated that it is an acidic PLA_2_. The PLA_2_ sequence contains conserved amino acid residues H^53^, Y^57^ and D^104^ as with other svPLA_2_, and in addition, D^49^ (Figure 5; positions in alignment figure: 53, 57, 104, 54, respectively) which is critical for its enzymatic activity [80,81]. In the venom proteomics, the protein abundance of PLA_2_ expressed was relatively higher (~14% of total venom proteins) than the transcription level in the venom gland (~2.1% of toxin FPKM). Low expression levels of PLA_2_ in snake venom gland transcriptomics have been reported for a number of Asiatic cobras (for instance, *N. naja* [31], *Naja kaouthia* [27], *Naja sumatrana* [35]) and the king cobra, *Ophiophagus hannah* [34,82], despite showing high protein abundances of PLA_2_ in their venom proteomes. The discrepancy could be due to a lower rate of PLA_2_ gene transcription, or a longer half-life of the PLA_2_ transcripts that allows prolonged protein translation. The protein abundance of PLA_2_ in NN-SL venom is consistent with that reported in most cobra venom proteomes (ranging from 11–21% of total venom proteins) [7,8,29,38,39], except for those in the *Uraeus* subgenus (African non-spitting cobras) whose venoms contain no or negligible amount of PLA_2_ [83,84]. 

The acidic PLA_2_ in cobra venoms generally have low toxicity (*i.v.* LD_50_ >10 mg/kg in mice) [54,55,63,85], but probably serve ancillary functions through synergistic interaction with other venom proteins such as WTX [86], CTX [87] and SVMP [88]. A recent study also demonstrated the synergistic action of upregulated PLA_2_ and CTX in eliciting pain for defense in three lineages of spitting cobras [89]. While *N. naja* is a non-spitting cobra, its PLA_2_ may exhibit similar potentiating activity with the CTX abundantly expressed in its venom, thereby enhancing the local tissue toxicity in envenomation. 

### 2.6. Comparison of Naja naja Venomics and Implication on Antivenom Treatment for Cobra Bite Envenomation

**Figure 6:**
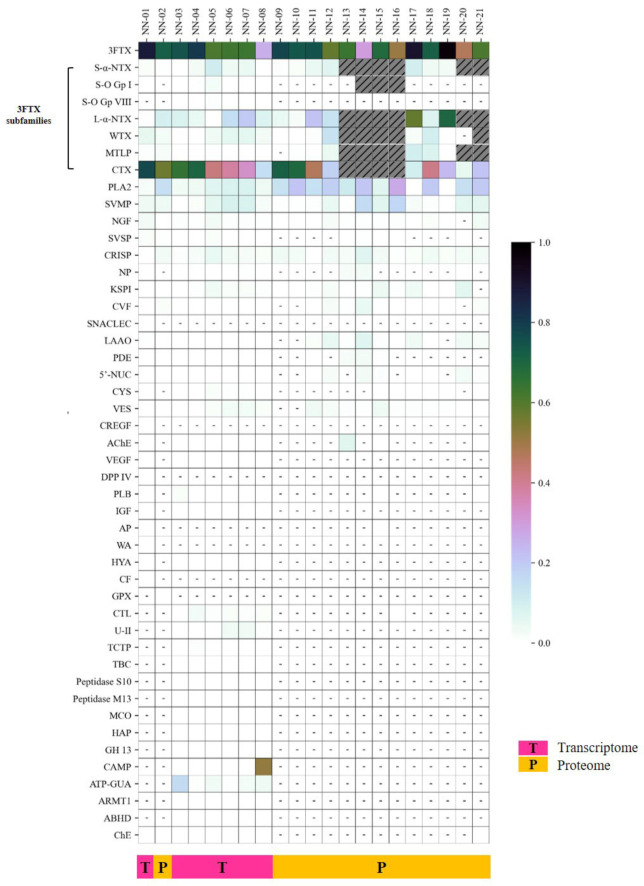
Heatmap of the comparative expression profiles of toxin families derived from the venom-gland transcriptome and venom proteome of *N. naja* from different geographical locales. The quantitative abundances were normalized on a scale of 0–1.0 for comparison purposes across different specimens, where 1.0 depicts 100% of relative abundance, as reported in respective studies. Unreported/undetected genes or proteins were denoted in dashed lines and unsorted subtypes (mainly 3FTX) were indicated with grey stripes. Details are available in Supplementary File S3. NN-01 and NN-02 represent the relative abundance ratio of toxin families expressed in the transcriptome and proteome of NN-SL (Colombo, Sri Lanka) in the current study, respectively. NN-03 and NN-04 correspond to the relative abundance ratio of toxin families expressed in the venom gland of *N. naja* specimens from Kerala, India, while NN-05 to NN-08 refers to the samples from the Kentucky Reptile Zoo [31]. NN-09 and NN-10 correspond to the relative abundance ratio of *N. naja* venom proteins from Sri Lanka and India, respectively [7]. NN-11 corresponds to the relative abundance ratio of *N. naja* venom proteins from Pakistan (Sindh) [8]. NN-12 corresponds to the relative abundance ratio of *N. naja* venom proteins from Southern Punjab, Pakistan [44]. NN-13 corresponds to the relative abundance ratio of *N. naja* venom proteins from West Bengal, India [26]. NN-14 corresponds to the relative abundance ratio of *N. naja* venom proteins from Tamil Nadu, India [40]. NN-15 and NN-16 correspond to the relative abundance ratio of *N. naja* venom proteins from Western Maharashtra [93]. NN-17, NN-18 and NN-19 correspond to the relative abundance ratio of *N. naja* venom proteins from Indian Punjab, Rajasthan and West Bengal [6]. NN-20 corresponds to the relative abundance ratio of *N. naja* venom proteins from Maharashtra, India [94]. NN-21 corresponds to the relative abundance ratio of *N. naja* venom proteins from West Bengal, India [30]. Abbreviations: 3FTX, three-finger toxins; S-α-NTX, short chain α-neurotoxin; S-O Gp I, short chain orphan group I, S-O Gp VIII, short chain orphan group VIII; L-α-LNTX, long chain α-neurotoxin; WTX, weak neurotoxin; MTLP, muscarinic toxin-like protein; CTX, cytotoxin/cardiotoxin; PLA_2_, phospholipase A_2_; SVMP, snake venom metalloproteinases; NGF, nerve growth factor; SVSP, snake venom serine protease; CRISP, cysteine-rich secretory protein; NP, natriuretic peptide; KSPI, Kunitz-type serine protease inhibitor; CVF, cobra venom factor; SNACLEC, snake C-type lectins; LAAO, L-amino acid oxidase; PDE, phosphodiesterase; 5’-NUC, 5’-nucleotidase; CYS, cystatin; VES, vespryn; CREGF, cysteine-rich with EGF domain-like; AChE, acetylcholinesterase; VEGF, vascular endothelial growth factor; DPP IV, dipeptidylpeptidase IV; PLB, phospholipase B; IGF, insulin-like growth factor; AP, aminopeptidase; WA, waprin; HYA, hyaluronidase; CF, coagulation factor; GPX, glutathione peroxidase; CTL, C-type lectin; U-II, urotensin-II; TCTP, translationally-controlled tumor protein; TBC, Type-B carboxylesterase; MCO, multicopper oxidase; HAP, histidine acid phosphatase; GH 13, glycosyl hydrolase 13; CAMP, cathelicidin antimicrobial peptide; ATP-GUA, ATP: guanido phosphotransferase; ARMT1, ARM-transferase; ABHD, AB hydrolase; ChE, cholinesterase.

Intra-species variation in snake venom composition is associated with differential venom toxicity and variable antivenom efficacy [23,90,91,92]. Across many studies reported for *N. naja* venom composition, the venom samples were typically sourced from various locales and assayed with different approaches, and therefore it is challenging for meaningful interpretation of the differential expression of the diverse toxins therein. To address this, we compiled data of *N. naja* venom proteomes and transcriptomes derived from the current work and previous studies, and the variations were illustrated as shown in Figure 6 for comparison purposes across different specimens, where the quantitative abundances were normalized on a scale of 0–1.0. 

We found that a total of 40 protein families with medical and toxinological relevance have been variably reported from the venom-gland transcriptomics and venom proteomics of *N. naja* specimens sourced from Sri Lanka (NN-01 and NN-02, Colombo region for current work; NN-09, unspecified locality [7]), India (NN-03 and NN-04, Kerala [31]; NN-10, Rajasthan and Gujarat [7]; NN-13, West Bengal [26]; NN-14, Tamil Nadu [40]; NN-15 and NN-16, Maharashtra [93]; NN-17, Punjab; NN-18, Rajasthan; NN-19, West Bengal [6]; NN-20, Maharashtra [94]; NN-21, West Bengal [30]), Pakistan (NN-11, related to Sindh [8]; NN-12, Punjab [44]) and the USA (NN-05 to NN-08, in Kentucky Reptile Zoo, supposedly originated from India or a captive-bred of Indian lineage). Four toxin families, 3FTX, PLA_2_, SVMP and CRISP, were detected and reported across all 21 specimens of *N. naja*, while other toxin families were much lowly expressed and not consistently present in all specimens. Of all toxins profiled, the 3FTX were the most highly expressed in all specimens irrespective of geographical origins, except in specimen NN-08 sourced from the Kentucky Reptile Zoo with cathelicidin antimicrobial peptide (CAMP) being the most abundantly expressed [31] (Figure 6). At the subproteome level, the 3FTX subtypes were variably detected. CTX were generally the major 3FTX expressed across all specimens while the content of lethal alpha-neurotoxins varied geographically. The alpha-neurotoxins were relatively higher in expression (abundance ratio of ~0.3–0.7 based on a scale of 0–1.0 per Figure 6) in the majority of *N. naja* specimens from the northern and eastern parts of the Indian subcontinent including Pakistan (NN-11 and NN-12) [8,44], Indian Punjab (NN-17) and West Bengal (NN-19) [6] compared to specimens from the southern part of South Asia, i.e., Kerala in South West India (NN-03 and NN-04, ~0.08) [31] and Sri Lanka (NN-01 and NN-02, present study; NN-09, [7]) (abundance ratio of < 0.2). The CTX expressions in the southern populations were, however, markedly higher (abundance ratio >0.6) than those in the northern and northeastern parts. With exceptions, specimens from Rajasthan and Gujarat in West India (NN-10 and NN-18) showed a relative higher CTX expression (abundance ratio of ~0.7) in contrast to their lowly expressed alpha-neurotoxins (abundance ratio of ~0.1) [6,7]. 

**Figure 7:**
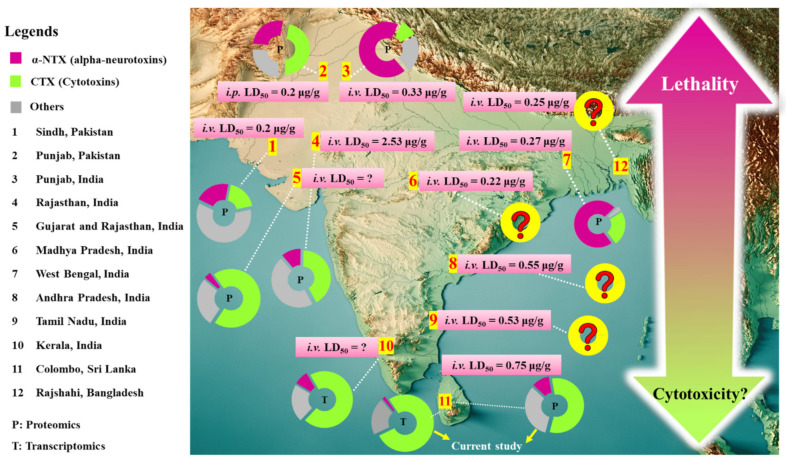
Comparison of the relative expression levels of alpha-neurotoxins and cytotoxins reported in the venom proteomes and/or venom-gland transcriptomes of *Naja naja* for snake populations from different localities in South Asia, including Pakistan, India, Sri Lanka (current work) and Bangladesh. Values of intravenous median lethal doses of the venoms were included where applicable, based on available references [6,8,44,107] except for locality #8 (Tamil Nadu, India, unpublished data from authors). Details of toxin abundances sorted were provided in Supplementary File S3.

The finding, in general, implies a venom phenotypic dichotomy characterized by differential expression of alpha-neurotoxins/cytotoxins in *N. naja* venom, and a somewhat imperfect distributional pattern of these venom phenotypes along the north–south axis of the Indian subcontinent including Sri Lanka (Figure 7). In brief, the Sri Lankan *N. naja*, representing the southernmost dispersed population exhibited a venom phenotype characterized by a lower alpha-neurotoxin abundance in exchange for a relatively higher CTX abundance, as opposed to that observed in the northern lineages from Pakistan, Indian Punjab and West Bengal [6,8,44]. Similar patterns of toxin compositional dichotomy within closely related species have been found among evolutionarily distant snake lineages from Elapidae (kraits, sea snakes, coral snakes) [95,96,97,98,99,100], and Viperidae (rattlesnakes, Russell’s vipers, etc.) [18,91,101,102], suggesting that intra-species venom phenotypic dichotomy might be more common than previously thought, and underscoring its impact on the effectiveness of antivenom treatment. The venom divergence within *N. naja* that follows a biogeographical pattern of north–south distribution implies local adaptation of the species to novel ecological niches. This results in diverging phenotypes that are shaped by the frequency of use and advantage of one toxin class/protein subtype over another as the species distributes southward. The phylogeographical factor alone, however, may not have represented the major driving force of such intra-species venom variation, and other evolutionary paths responsible for generating such venom variability should be studied with more extensive sampling across the biogeographical range of *N. naja*. Further research should also aim to profile the venom proteome of *N. naja* populations from biologically significant areas such as Tamil Nadu (from where snake venoms are sourced for the production of most Indian antivenoms), Kerala (for the unique shola ecosystem in the Western Ghats with high biological diversity and endemism), Maharashtra and Rajasthan (for correlation of alpha-neurotoxin/cytotoxin abundances and venom LD_50_). Earlier, the venom proteome of cobra from Maharashtra was shown to contain as high as 41.8% of neurotoxic 3FTX (alpha-neurotoxins clustered with various atypical and unconventional 3FTX), and very little cytotoxic 3FTX (CTX) at only 5.4% of total venom protein [94]. The venom seems to exhibit atypical toxicity that is highly varied from other cobra species, and is therefore worthy of investigation. Its very high content of neurotoxic 3FTX (reported at 41.8% of protein abundance) apparently did not contribute proportionally to the venom-induced neurotoxicity, judging from its reported *i.v.* LD_50_ of 0.73 µg/g which indicates modest lethality for a cobra venom [39]. The abundance of cytotoxic 3FTX reported was exceptionally low (compared to well-established CTX composition in cobra venoms), notwithstanding the fact that cobra bite envenomation in Maharashtra produces local envenoming effect of tissue toxicity [103,104]. On the other hand, *N. naja* venom from Rajasthan was shown to have extremely low lethality (*i.v.* LD_50_ = 2.53 µg/g) [6]. Based on the study, the Rajasthani *N. naja* venom is perhaps the least lethal amongst all cobra species known, despite having a moderate amount of alpha-neurotoxins at 11.4% (note: reported at 30.1% as “neurotoxic 3FTX”) [6]. This protein abundance of alpha-neurotoxins is at par with *N. atra* from Zhejiang (China, 11.2%) and *N. kaouthia* from Vietnam (9.2%) and Guangxi (China, 13.4%), whose LD_50_ values were established in the range between 0.68 and 0.90 µg/g, in mice [23,43,105]. Clinically, cobra bite in Rajasthan significantly contributed to 53.6% of 138 envenomation cases and was associated with a high mortality of 61.5% in a 2-year hospital-based epidemiological study, where patients were found to manifest neurological deficits with diminished oxygen saturation suggestive of prominent paralysis [106]. The intriguing though somewhat conflicting findings between previously reported venom proteomes, venom toxicity study and clinical manifestation of envenoming warrant a revisitation to resolve the discrepancies.

The comparative venomics to date suggests a high degree of 3FTX evolvability in *N. naja*, and the consequent variability in toxin expression that bears toxicological correlations with effects attributed to the venom proteins and their neutralization. In mice, the lethal potencies of various cobra venoms have been shown to correlate with the content of alpha-neurotoxins in the venom [39]; here, we observed a similar trend even within the same species of *N. naja* from different locales. For *N. naja* of the northern distribution (NN-11: Sindh, Pakistan; NN-12: Punjab, Pakistan; NN-17, Punjab, India; NN-19; West Bengal, India), whose alpha-neurotoxins are more abundant, the venoms elicited rapid neurotoxic effect and high lethality with low median lethal doses (*i.v.* or *i.p.* LD_50_) ranging from 0.2 µg/g to 0.3 µg/g [6,8,44] (Figure 7). Similarly, the venom of *N. naja* from Rajshahi, Bangladesh has a low *i.p.* LD_50_ of 0.25 µg/g [107], although its venom proteomics has not been studied to reveal the quantitative abundance of alpha-neurotoxins in the venom. On the other hand, the venom of NN-SL tested in the present study (representing the southernmost dispersal of *N. naja*) has an *i.v.* LD_50_ of 0.75 µg/g in mice, which is comparable to values previously reported for Sri Lankan cobras (0.67 mg/kg [12]; 1.13 mg/kg [22]) and is consistent with the relatively lower abundance of alpha-neurotoxins in its venom. Meanwhile, the venoms of *N. naja* from the south (Tamil Nadu) and south-east (Andhra Pradesh) of the Indian subcontinent have *i.v.* LD_50_ values of 0.53 µg/g (unpublished) and 0.55 µg/g [6], respectively, suggesting that the venoms likely contain a moderate amount of alpha-neurotoxins that is intermediate between the northern and the Sri Lankan populations. The finding indicates that the lethality of *N. naja* cobra venom varies with the expression and the abundance of α-NTX in the venom proteome, with an observable shift in potency decreasing as the species distributes southward (Figure 7). A trend of increasing cytotoxins appears to accompany the shift, though clinically local tissue necrosis caused bty cobra bite is common throughout the region.

Consequently, the variable expression of the principal toxins (α-NTX and CTX) is anticipated to influence the effectiveness of antivenom treatment for envenomation caused by *N. naja* in different geographical areas. This study further investigated the neutralization activity of VPAV, a commonly used Indian polyvalent antivenom in Sri Lanka, against the lethality of NN-SL venom at a challenge lethal dose of 2.5 LD_50_. The antivenom moderately neutralized the NN-SL venom lethality with a median effective dose (ED_50_) of 35 µL of antivenom, a dose at which half of the challenged mice survived. The neutralization potency of VPAV against NN-SL venom was determined accordingly as 0.74 mg venom per mLantivenom, and compared with the neutralization activities of Indian antivenoms previously reported for *N. naja* of various locales (Table 4). The data from the current study fell within the range of potency (Potency = 0.3–0.7 mg/mL) reported for commercially available Indian antivenom products, namely VINS polyvalent antivenom, Bharat antivenom and Premium Serums antivenom, in neutralizing the lethality of cobra venoms from various locales in South Asia [6,8,12,22,54,94]. Based on the neutralization potency, a vial of antivenom reconstituted in 10 mLwater should be able to neutralize approximately 7 mg of the NN-SL cobra venom. Considering an adult cobra snake can deliver more than 50 mg of venom in a bite, it is estimated that approximately 10 vials of antivenom and above would be required for effective treatment. Clinically, neurotoxic envenomation cases caused by cobra bite in Sri Lanka were treated with considerably high doses of Indian antivenoms ranging from 8–30 vials [15,20]. Meanwhile, antivenoms generally have limited effectiveness against the local envenoming effect of cobra bite that causes tissue necrosis [13]. A recent study demonstrated that the Indian antivenoms (VINS and Bharat) were less effective in reversing the local myotoxicity of Sri Lankan *N. naja* venom tested in an *in vitro* chick biventer nerve-muscle preparation [17]. In fact, the neutralization effect of antivenom against venom-induced tissue destruction in envenomation caused by most Asiatic cobra bites is known to be weak, as shown in humans clinically and assessed in laboratory animals [56,108,109,110]. Overall, the finding underscores the need for antivenom products with higher efficacy to neutralize the venom-induced toxicity caused by cobra bite envenomation in Sri Lanka. The existing antivenom manufacturing process based in India may be improved by reformulation of immunogen based on the venomics of *N. naja* from various locales. The principal toxins specific to different locales including Sri Lanka can be incorporated into the immunogen mixture to optimize the immunization of horses during antiserum production [111,112,113].

## 3. Conclusions

Incorporating *de novo* venom-gland transcriptomics and venom proteomics, the present study elucidated the complexity of the Sri Lankan *N. naja* (NN-SL) venom composition. A total of 22 toxin gene families unique to NN-SL were uncovered, and 12 of these gene families were translated into various protein forms detected in the venom proteome. The 3FTX were the most abundantly expressed, while variation in the 3FTX subtypes, in particular alpha-neurotoxins (α-NTX) and cytotoxins (CTX), correlated with the differential venom toxicity of *N. naja* from various locales. The NN-SL which represented the southernmost dispersal of *N. naja*, has a relatively lower content of venom α-NTX and consequently a higher LD_50_ of its venom, in contrast to the far northern and northeastern populations whose venoms contain more α-NTX and show higher lethality. Despite the venom variability, the Indian polyvalent antivenom moderately neutralized the lethality induced by the Sri Lankan cobra venom. Its neutralization potency was limited, and thus underscoring the need for an improved antivenom product with higher efficacy and broader geographical utility in the region.

## 4. Materials and Methods

### 4.1. Snake Venom, Venom Gland Preparation and Antivenom

The cobra, *N. naja* was an adult snake originated from Colombo, Sri Lanka. The venom was collected by inducing the snake to bite through a container covered with a sterile plastic film, and the venom was lyophilized and stored at −20 °C until use. The snake was rested for four days for the transcription process to continue in its venom glands for four days. This was followed by euthanasia of the snake and quick removal of the venom gland, which was sectioned and preserved in the RNAlater^®^ solution (Ambion, TX, USA). The procedures of venom milking and tissue collections were carried out according to the protocol and guideline approved by the Institutional Animal Use and Care Committee (IACUC) of the University of Malaya, Malaysia (approval number: #2013-11-12/PHAR/R/TCH). Indian VINS polyvalent antivenom (VPAV, batch no: 01AS12041) used in the current study was produced from horse antisera hyperimmunized against the venom of Indian “Big Four”, i.e., *Daboia russelii*, *Naja naja*, *Bungarus caeruleus* and *Echis carinatus*. The antivenom was reconstituted in 10 mLultrapure water as per manufacturer’s instruction prior to use.

### 4.2. Chemicals and Materials

All chemicals and materials used were of analytical grade. Ammonium bicarbonate, dithiothreitol (DTT) and iodoacetamide (IAA) were purchased from Sigma-Aldrich (St. Louis, MO, USA). MS grade trypsin protease, Spectra™ Multicolor Broad Range Protein Ladder (Catalog number: 26634, 10 to 260 kDa), and HPLC grade solvents were purchased from Thermo Scientific™ (Pierce™, Rockford, IL, USA). Millipore ZipTip^®^ C_18_ Pipette Tips were obtained from Merck KGaA (Darmstadt, Germany). Other chemicals and reagents of analytical grade were purchased from Sigma-Aldrich (St. Louis, MO, USA).

### 4.3. RNA Extraction and Purification

The dissected venom gland tissue was homogenized under sterile conditions in a 1 mLglass homogenizer submerged with TRIzol solution (Invitrogen, Carlsbad, CA, USA). This was followed by the addition of chloroform and treated with RNA-free DNAase I (Thermo Fisher Scientific, Waltham, MA, USA) to separate RNA from cellular debris and residual DNA. The RNA was isolated with isopropyl alcohol and ethanol precipitation. Polyadenylated mRNA was subsequently purified with oligo (dT) magnetic beads according to the manufacturer’s protocol (Illumina, San Diego, CA, USA). The quality of the purified mRNA was subsequently checked using the Agilent 2100 Bioanalyzer (Agilent Technologies, Waldbronn, Germany).

Enriched poly(A)+ mRNA isolated from the total venom-gland RNA was used for cDNA construction. The isolated mRNA was fragmented into short fragments, which acted as templates for cDNA synthesis [114]. Random hexamer-primer (N6) was used to synthesis the first-stranded cDNA as input materials, using second strand buffers, dNTPs, RNase H and DNA polymerase I. From these cDNA, a paired-end library was synthesized using the Genomic Sample Prep kit (Illumina, San Diego, CA, USA), according to the manufacturer’s instructions. The cDNA fragments generated were purified with QIAquick PCR extraction kit (Qiagen, Valencia, CA, USA) and dissolved in elution buffer for end repair and the addition pf poly(A) to aid in the subsequent ligation of Illumina adaptors that contain a single thymine (T) base overhang at the 3’ ends. Following the ligation, these cDNA fragments were amplified via polymerase chain reaction (PCR) electrophoresed on a 1.5–2% TAE (Tris-base, acetic acid and EDTA) agarose gel. From the gel, suitable fragments (200–700 bp) were selected as templates for subsequent PCR amplification. Sequencing of the amplified samples library was achieved in a single lane on the Illumina HiSeq™ 2000 platform (Illumina, San Diego, CA, USA) with 100-base-pair, paired-end reads.

### 4.4. Raw Sequenced Reads and De Novo Transcriptome Assembly

Sequenced data generated from Illumina HiSeq™ 2000 were transformed by base calling into sequence data, called the raw reads and stored in a FASTQ format. Prior to transcriptome assembly, raw reads were filtered to generate clean reads as part of the quality control process in the pre-analysis stage [115]. This involved the removal of (i) adaptor; (ii) reads with >5% of unknown nucleotides or (ii) low-quality reads with > 20% of low-quality bases (determined as base quality <10).

*De novo* transcriptome assembly was performed using short reads assembly program, Trinity (version 2.0.6) [116,117]. Three independent software modules, i.e., Inchworm, Chrysalis and Butterfly, comprised the Trinity program, were sequentially applied to process the large volumes of RNA-seq reads based on the algorithm of de Bruijn graph construction. The process began by aligning k-mers (k = 25), and sequence reads with a certain length of overlap were joined to form linear contigs. The sequence reads were mapped back onto contigs, and by referring to paired-end reads, contigs from the same transcript, as well as the distances between them were determined. The contigs were then partitioned into clusters, each of which carried a complete set of de Bruijn graphs as a representation of the transcriptional complexity at a given gene or locus. The graphs were independently processed to obtain full-length transcripts for alternatively spliced isoforms and to tease apart transcripts that corresponded to paralogous genes. The clean read Q20 percentage, a point of reference for quality control assessment was obtained as a benchmark for successful *de novo* assembly of the transcriptome.

### 4.5. Transcripts Clustering and Functional Annotation

The transcript sequences generated from Trinity (known as Unigenes) were further processed for sequence splicing and the removal of redundancy using TGI clustering tools (TGICL) (version 2.1) to acquire the longest possible length of non-redundant (NR) transcripts. The transcripts were then subjected to family clustering into two classes of transcripts: (a) clusters, with a prefix CL and the cluster ID behind as contig; (b) singletons, ID with a prefix of Unigene. In each cluster, there were several transcripts with sequence similarities among them being >70% while the singletons “Unigenes” lack of overlapping with other fragments at the given stringency. In brief, the value 70% was used to categorize the assembled sequences based on similarity; sequences similar to each other (may or may not be homologous as having >70% similarity) were grouped under a cluster comprising various contigs.

Subsequently, transcript Unigenes were aligned using BLASTx to NCBI non-redundant (NR) protein database, with a cut-off value of E < 10_−5_. The proteins with the highest rank in the BLASTx results were referred to determine the coding region sequences of the Unigenes, followed by translation into amino acid sequences using standard codon table. Hence, both nucleotide sequences (5′–3′) and amino acid sequences of the Unigene-coding regions were acquired. To remove redundancy from each cluster, the longest sequence in each cluster was chosen as the transcript, and the length of the scaffold was extended based on overlapping sequences using Phrap assembler (release 23.0) (http://www.phrap.org) (accessed on 14 September 2020). The distribution lengths of contigs, scaffolds and Unigenes were calculated, and the N50 length (assembly quality indicator) was set at >500 for success assembly.

### 4.6. Quantification of Transcript Abundance

Clean reads were aligned to Unigene using Bowtie2 [118]. The transcript abundances were calculated using RNA-seq with expectation maximization (RSEM) software [119]. Fragments per kilobase of exon model per million reads mapped (FPKM) were used to determine the transcript abundance for the identified genes [120]. FPKM is the summation of normalized read counts based on gene length and the total number of mapped reads. The data was obtained using RSEM tool in conjunction with Trinity based on a computational formula:

(1) $$\mathrm{FPKM}\;\mathrm{of}\;\mathrm{gene}\;A=\frac{10^{6}\;B}{NC\;/10^{3}}$$

FPKM is the expression of gene *A*; *B* is the number of fragments/reads which are aligned to gene *A*, *N* is the total number of fragments/reads that are aligned to all genes; *C* is the base number in the coding sequence of gene *A*.

### 4.7. Categorization of Transcripts

The *de novo* assembled transcripts were subjected to BLASTx search to obtain the closest resembling sequences from the NR protein database for further classification based on functional annotations. The transcripts were then sifted to remove those with an FPKM value of less than 1, followed by categorization into three groups: “toxins”, “non-toxins” and “unidentified” (Supplementary File S1) [33,35]. “Toxin” transcripts were recruited by toxin-related keyword searches against the annotated transcripts. “Non-toxin” and “unidentified” groups contain transcripts of cellular proteins or house-keeping genes and transcripts that could not be identified, respectively. The redundancy of gene expression was determined by dividing the total FPKM of each group by the total number of transcripts in the respective group of transcripts [27]. In the “toxin” group, the amino acid sequences were used to further validate the toxin identity through BLASTp suite (Basic Local Alignment Search Tool-Protein) in the Uniprot (Universal Protein Resource Knowledgebase) database platform. The transcripts were searched against Serpentes database (taxid: 8570) and validated based on the lowest E-score value with the highest percentage of sequence similarity (updated as of September 2019). The annotation was manually filtered to proteins specific for elapids only.

### 4.8. Multiple Sequence Alignment

Multiple sequence alignment was conducted using Jalview software (version 2.11.1.4) [121,122] and aligned using MUSCLE (multiple sequence comparison by log-expectation) program [123]. Sequences of related species used in multiple sequence alignment were retrieved from UniprotKB depository (http://www.uniprot.org/) (accessed on 14 September 2020). The selection was based on their relevance to the toxins in comparison to elucidate the similarity and variation as well as conserved regions of the sequences. 

### 4.9. Correlation Analysis

The correlation of the toxin gene expression of each family (from venom-gland transcriptome) and the protein abundance of the corresponding toxin family (from venom proteome) was analyzed by Spearman’s correlation coefficient and constructed using GraphPad Prism 6 (San Diego, CA, USA). The significance of the correlation was estimated by the coefficient of determination (r) at a cutoff value of *p* < 0.05. 

### 4.10. Heatmap of Differentially Expressed Toxins Genes and Proteins

A heatmap of relative abundance pattern in the differentially expressed toxin genes and venom proteins of *N. naja* from various geographical locales were generated with the custom scripts of Python version 3.8.5. The dataset was derived from previous proteomic and transcriptomic studies on *N. naja* from Sri Lanka, India and Pakistan [6,7,8,26,30,31,40,44,93,94] and the current work on NN-SL from Sri Lanka (dataset available in Supplementary File S3). The relative abundances were depicted by color scheme ranging from low (white) to high (black) intensity, based on a scale of 0 to 1.0 where 1.0 represented 100% of relative abundances of transcript or protein, as reported in the respective studies. Unreported or undetected genes and proteins were denoted with dashed lines, and unsorted subtypes (3FTX mainly) were indicated with grey stripes.

### 4.11. Reverse-Phase High-Performance Liquid Chromatography (RP-HPLC)

*Naja naja* venom (3 mg) was reconstituted in 200 µL ultrapure water and subjected to C-18 RP-HPLC using Shimadzu LC-20 AD HPLC system (Shimadzu, Kyoto, Japan). The LiChrospher^®^ WP 300 C-18 column (5 µm pore size) was pre-equilibrated with Eluent A (0.1% TFA in water) and eluted with Eluent B (0.1% TFA in ACN) using a linear gradient of 5% B for 10 min, 5–15% B over 20 min, 15–45% B over 120 min and 45–70% B over 20 min. The flow rate was set to 1 ml/min. The fractionation was monitored at 215 nm absorbance, and the resulting peaks were collected, lyophilized and stored at −20 °C prior to use. 

### 4.12. Sodium Dodecyl Sulfate-Polyacrylamide Gel Electrophoresis (SDS-PAGE)

The fractions collected from RP-HPLC were subjected to SDS-PAGE according to Laemmli [124]. Approximately 20 µg of venom protein fractions were loaded into the wells, and fractionation was performed with 15% SDS-PAGE under reducing condition at 90 V for 2 h. Thermo Scientific Spectra™ Multicolor Broad Range Protein Ladder (10–260 kDa) was used as calibration standards. The gels were stained with Coomassie Brilliant Blue R-250, and protein bands were visualized and scanned using ImageScanner III (GE Healthcare, Danderyd, Sweden). 

### 4.13. In-Gel Tryptic Digestion and Tandem Mass Spectrometry

Protein bands of interest were excised into small gel pieces (~2 mm), reduced with dithiothreitol, alkylated with iodoacetamide and digested with MS grade Pierce trypsin protease according to the manufacturer’s protocol (Thermo Scientific™ Pierce™, Rockford, IL, USA). Digested peptides were extracted and desalted using Millipore ZipTip^®^ C_18_ Pipette Tips.

The tryptic digested peptides were then subjected to MALDI TOF/TOF using AB SCIEX TOF/TOF™ 5800 System Proteomic Analyzer (AB SCIEX, Framingham, MA, USA) equipped with neodymium: yttrium-aluminum-garnet laser (laser wavelength was 355 nm). Samples (0.5 µL) were mixed with alpha-cyano-4-hydroxycinnamic acid matrix (0.5 µL) and spotted on OPTI-TOF™ LC/MALDI insert plate (123 mm × 81 mm). For MS mode, peptide mass maps were acquired in positive reflection mode at 1000 laser shots per spectrum at 1 kV with a mass range of 800–4000 *m*/*z*. The MS/MS peak detection criteria were a minimum signal-to-noise ratio of 100. The raw mass spectra acquired were exported to AB SCIEX ProteinPilot™ Software and search against a customized database consisting of the non-redundant protein sequence database from NCBI (taxonomy: Serpentes; taxid: 8570) and our in-house transcripts database. MS peak filter mass range 800–4000 *m*/*z* was applied. Precursor and fragment mass tolerances were set to 100 ppm and 0.2 Da and allowing one missed cleavage. Oxidation (M) was set as a variable modification, and carbamidomethylation (C) was set as a fixed modification. The protein score intervals (C.I.) above 95% were considered as confident identification.

The unidentified peptide samples from MALDI TOF/TOF were subjected to nano-electrospray ionization MS/MS using Agilent 1200 HPLC-Chip/MS Interface, coupled with Agilent 6550 Accurate-Mass Q-TOF LC/MS system. Samples were loaded in a large capacity chip 300 Å, C18, 160 nl enrichment column and 75 µm × 150 mm analytical column (Agilent part no. G4240-62010) with a flow rate of 4 µL/ min from a capillary pump and 0.4 µL/min from a nano pump of Agilent 1200 series. The injection volume was 2 µL per sample, and the mobile phase was 0.1% formic acid in water (Solution A) and 0.1% formic acid in 100% acetonitrile (Solution B). The gradient applied was: 5–50% B for 11 min, 50–70% B for 4 min and 70% B for 3 min using Agilent 1200 series nano-flow LC pump. Ion polarity was set to positive ionization mode. Drying gas flow was 11 L/min and drying gas temperature was 290 °C. Fragmentor voltage was 175 V and the capillary voltage was set to 1800 V. Spectra was acquired in a MS/MS mode with an MS scan range of 200–3000 *m*/*z* and MS/MS scan range of 50–3200 *m*/*z*. Precursor charge selection was set as doubly charge state and above with the exclusion of precursors 1221.9906 *m*/*z* (z = 1) and 299.2944 (z = 1) set as reference ions. Data were extracted with MH^+^ mass range between 50 and 3200 Da and processed with Agilent Spectrum Mill MS Proteomics Workbench software packages. Carbamidomethylation of cysteine was set as a single modification. The peptide finger mapping was modified to specifically search against a customized database consisting of the non-redundant protein sequence database from NCBI (taxonomy: Serpentes; taxid: 8570) and our in-house transcripts database. Protein identification were validated with the following filters: protein score >11, peptide score >10 and score peak intensity (SPI) >70%. The result for “Distinct peptide” identification of greater than one is considered significant. 

### 4.14. Estimation of Protein Relative Abundance

The protein relative abundance was estimated according to the method used earlier in proteomic studies [8,23]. In brief, the relative abundance of venom fractions was estimated by peak area under curve using Shimadzu LC Solution Software (Shimadzu, Kyoto, Japan). The fractions that show a homogenous protein band in SDS-PAGE was directly implemented with the relative abundance obtained from peak area under curve (AUC) of chromatogram. For the fractions with various protein bands in SDS-PAGE, the relative abundance of individual bands in a fraction were estimated by gel densitometry using Thermo Scientific^™^ Pierce^™^ MyImageAnalysis™ Software (Thermo Fisher Scientific, Waltham, MA, USA). The relative abundance (in percentage) of these individual protein bands was the product of multiplying the peak area under the curve (AUC) with gel densitometry of protein bands. The relative abundances were then accumulated according to the protein family. 

### 4.15. Determination of Venom Lethality and Neutralization by Antivenom

The study was carried out by intravenous administration of various doses of venom (four doses of venom tested) via the caudal vein into the ICR albino mice (20–25 g, n = 4 per dose). The mice were allowed free access to food and water ad libitum. The survival ratio of mice was recorded at 24 h post-injection for the determination of intravenous median lethal dose (*i.v.*, LD_50_) at which 50% of the tested animal were dead. 

For antivenom neutralization, the study was conducted as previously described in [125]. In brief, a challenge dose of venom at 2.5 times LD_50_ dissolving in saline water was pre-incubated with various dilutions of antivenom (4 dilutions of antivenom tested, 15 μL, 35 μL, 75 μL and 100 μL) at 37 °C for 30 min. The venom–antivenom mixture was then injected intravenously into the mice (20–25 g, n = 4 per dose). The mice were allowed free access to food and water *ad libitum*. The survival ratio of the mice was recorded at 24-h post-injection for the determination of median effective dose (ED_50_) of the antivenom against the venom. ED_50_ was defined as the antivenom dose (µL) that prevented death in 50% of the tested animals. 

Both LD_50_ and ED_50_ values and 95% C.I. were calculated using the Probit analysis method using Biostat 2009 software [126]. The neutralization efficacy was also expressed as Potency, (P), defined as the amount of venom (mg) completely neutralized per unit volume of antivenom (ml) [127,128].

### 4.16. Supporting Data

Sequencing data from the *de novo* venom-gland transcriptomics of *Naja naja* was deposited in National Centre for Biotechnology Information (NCBI) Sequence Read Archive (http://submit.ncbi.nlm.nih.gov/subs/sra/) (submitted on 28 June 2021) under SRA accession: PRJNA741865). The mass spectrometry proteomics data have been deposited to the ProteomeXchange Consortium via the iProX partner repository (http://www.iprox.org) (accessed on 14 September 2020) [129] with dataset identifier PXD026735 (Subproject ID: IPX0003192001) (submitted on 16 June 2021).

## Figures and Tables

**Table 1:** Descriptive statistics of RNA-sequencing data for NN-SL venom gland transcriptome.

Parameter	Sequencing Output
Total raw reads	54,477,234
Total clean reads	51,595,008
Total clean nucleotides (nt)	4,643,550,720
Q20 (%)	98.50
N (%)	0.01
GC (%)	44.62
**Contigs assembly statistics**	106,637
Total length (nt)	28,724,569
Mean length (nt)	269
N50 length (bp)	361
**Unigenes assembly statistics**	53,595
Total length (nt)	25,919,321
Mean length (nt)	484
N50 length (bp)	676

Q20 is the proportion of nucleotides with quality value larger than 20; *N* percentage is the proportion of unknown nucleotides in clean reads; GC percentage is the proportion of guanidine and cytosine nucleotides among the total nucleotides; N50 is the shortest contig length to cover 50% of the transcriptome.

**Table 2:** Overview of toxin gene families and transcript abundances (%) in venom gland transcriptome of NN-SL.

Gene ID	Protein Family/ Subfamily	Annotated Accession (NCBI/Uniprot)	Species	Coverage ^A^ (%)	Transcript ^B^ Abundance (%)
**Three-finger toxins (3FTX)**					**87.54%**
**Short-chain 3FTX: Short α-NTX (SNTX)**					1.78%
NN-3FTX01	Short neurotoxin 2	P62376	*Hydrophis cyanocinctus*	13–79	0.11%
NN-3FTX02 *	Cobrotoxin	P60770	*Naja atra*	1–82 (98.80)	0.69%
NN-3FTX03 *	Cobrotoxin-b	P80958	*Naja atra*	1–82 (100)	0.97%
**Short-chain 3FTX: Orphan Group I**					0.61%
NN-3FTX04	Neurotoxin-like protein NTL2	Q9W717	*Naja atra*	1–75	0.61%
**Short-chain 3FTX: Muscarinic toxin-like protein (MTLP)**					1.37%
NN-3FTX05 *	Neurotoxin homolog NL1	Q9DEQ3	*Naja atra*	6–86 (94.19)	0.96%
NN-3FTX06	Muscarinic toxin-like protein 2	P82463	*Naja kaouthia*	1–46	0.41%
NN-3FTX07	Haditoxin	A8N286	*Ophiophagus hannah*	36–84	0.001%
**Short-chain 3FTX: Cytotoxin/cardiotoxin (CTX)**					77.33%
NN-3FTX08 *	Cytotoxin 5	Q98961	*Naja atra*	1–81 (100)	77.33%
**Long-chain 3FTX: Long α-NTX (LNTX)**					1.10%
NN-3FTX09 *	Long neurotoxin 7	O42257	*Naja sputatrix*	12–88 (97.78)	1.00%
NN-3FTX10	Long neurotoxin 1	Q8UW29	*Hydrophis hardwickii*	12–91	0.01%
NN-3FTX11	Long neurotoxin-like OH-31	Q53B55	*Ophiophagus hannah*	20–59	0.08%
NN-3FTX12	Long neurotoxin-like OH-31	Q53B55	*Ophiophagus hannah*	5–59	0.003%
**Non-conventional 3FTX: Weak neurotoxin (WTX)**					5.35%
NN-3FTX13	Probable weak neurotoxin NNAM1	Q9YGI2	*Naja atra*	15–86	5.35%
**Phospholipase A_2_ (PLA_2_)**					**2.08%**
NN-PLA01	Basic phospholipase A_2_	P00610	*Hydrophis schistosus*	23–119	0.01%
NN-PLA02	Basic phospholipase A_2_	P00610	*Hydrophis schistosus*	23–119	0.002%
NN-PLA03	Acidic phospholipase A_2_ 57	Q8UW31	*Hydrophis hardwickii*	51–135	0.01%
NN-PLA04 *	Acidic phospholipase A_2_ 2	P00597	*Naja kaouthia*	1–146 (100)	2.05%
NN-PLA05	Basic phospholipase A_2_ 73	Q8UW30	*Hydrophis hardwickii*	1–56	0.01%
**Snake venom metalloproteinase (SVMP)**					**3.80%**
NN-SVMP01	Scutatease-1	B5KFV7	*Notechis scutatus*	337–388	0.04%
NN-SVMP02	Zinc metalloproteinase mocarhagin	Q10749	*Naja mossambica*	502–568	0.03%
NN-SVMP03	Zinc metalloproteinase mocarhagin	Q10749	*Naja mossambica*	191–241	0.03%
NN-SVMP04	Zinc metalloproteinase mocarhagin	Q10749	*Naja mossambica*	143–195	0.03%
NN-SVMP05	Zinc metalloproteinase-disintegrin cobrin	Q9PVK7	*Naja kaouthia*	159–499	0.27%
NN–SVMP06	Zinc metalloproteinase-disintegrin cobrin	Q9PVK7	*Naja kaouthia*	159–600	0.16%
NN-SVMP07	Zinc metalloproteinase-disintegrin cobrin	Q9PVK7	*Naja kaouthia*	159–600	0.13%
NN-SVMP08	Zinc metalloproteinase-disintegrin atrase-B	D6PXE8	*Naja atra*	322–369	0.01%
NN-SVMP09	Zinc metalloproteinase-disintegrin atrase-A	D5LMJ3	*Naja atra*	456–607	0.10%
NN-SVMP10	Zinc metalloproteinase-disintegrin atrase-A	D5LMJ3	*Naja atra*	456–607	0.10%
NN-SVMP11	Zinc metalloproteinase-disintegrin atrase-A	D5LMJ3	*Naja atra*	456–607	0.15%
NN-SVMP12	Zinc metalloproteinase-disintegrin atrase-A	D5LMJ3	*Naja atra*	403–607	0.13%
NN-SVMP13	Zinc metalloproteinase-disintegrin atrase-A	D5LMJ3	*Naja atra*	403–607	0.17%
NN-SVMP14	Zinc metalloproteinase-disintegrin atrase-A	D5LMJ3	*Naja atra*	456–607	0.28%
NN-SVMP15	Zinc metalloproteinase-disintegrin atrase-A	D5LMJ3	*Naja atra*	1–229	1.25%
NN-SVMP16	Hemorrhagic metalloproteinase-disintegrin kaouthiagin	P82942	*Naja kaouthia*	6–64	0.03%
NN-SVMP17	Zinc metalloproteinase-disintegrin-like atragin	D3TTC2	*Naja atra*	527–613	0.80%
NN-SVMP18	Hemorrhagic metalloproteinase-disintegrin kaouthiagin	P82942	*Naja kaouthia*	130–201	0.07%
**Cysteine-rich secretory protein (CRISP)**					**0.35%**
NN-CRISP01 *	Natrin-1	Q7T1K6	*Naja atra*	1–239 (100)	0.35%
**Snake venom serine protease (SVSP)**					**1.56%**
NN-SVSP01 *	Serine protease harobin	Q5MCS0	*Hydrophis hardwickii*	1–265 (96.60)	1.56%
NN-SVSP02	Serine protease HTRA1	V8NGT0	*Ophiophagus hannah*	79–304	0.001%
NN-SVSP03 *	Serine protease 23	V8N8N4	*Ophiophagus hannah*	8–372 (98.12)	0.001%
**Cobra venom factor (CVF)**					**0.26%**
NN-CVF01	Cobra venom factor	Q91132	*Naja kaouthia*	1586–1642	0.13%
NN-CVF02 *	Cobra venom factor	Q91132	*Naja kaouthia*	1–1592 (96.96)	0.10%
NN-CVF03	Cobra venom factor	Q91132	*Naja kaouthia*	1197–1592	0.02%
NN-CVF04	Cobra venom factor	Q91132	*Naja kaouthia*	1–640	0.01%
**Kunitz-type serine protease inhibitor (KSPI)**					**0.50%**
NN-KSPI01 *	Putative Kunitz-type serine protease inhibitor	B2BS84	*Austrelaps labialis*	1–252 (98.81)	0.02%
NN-KSPI02 *	Kunitz-type protease inhibitor	U3FZD6	*Micrurus fulvius*	1–511 (99.61)	0.004%
NN-KSPI03	Protease inhibitor 3	C1IC51	*Walterinnesia aegyptia*	1–67	0.17%
NN-KSPI04	Kunitz-type serine protease inhibitor NACI	Q5ZPJ7	*Naja atra*	43–81	0.30%
**Phosphodiesterase (PDE)**					**0.08%**
NN-PDE01 *	Snake venom phosphodiesterase	A0A2D0TC04	*Naja atra*	21–850 (97.65)	0.08%
**5’-Nucleotidase (5’-NUC)**					**0.06%**
NN-NUC01 *	Ecto-5’-nucleotidase	A0A194AS98	*Micrurus tener*	1–569 (99.13)	0.05%
NN-NUC02 *	Ecto-5’-nucleotidase	A0A194AS98	*Micrurus tener*	1–536 (93.38)	0.008%
NN-NUC03 *	Ecto-5’-nucleotidase	A0A194AS98	*Micrurus tener*	1–536 (93.38)	0.004%
NN-NUC04	5’ nucleotidase	A6MFL8	*Demansia vestigiata*	134–559	0.001%
**Acetylcholinesterase (AChE)**					**0.01%**
NN-ACHE01 *	Acetylcholinesterase	Q92035	*Bungarus fasciatus*	1–566 (95.55)	0.004%
NN-ACHE02	Acetylcholinesterase	Q92035	*Bungarus fasciatus*	1–566 (95.55)	0.002%
**Hyaluronidase (HYA)**					**0.002%**
NN-HYA01	Hyaluronidase	V8PHI0	*Ophiophagus hannah*	26–71	0.001%
NN-HYA02	Hyaluronidase	A0A2D4JS30	*Micrurus lemniscatus lemniscatus*	89–208	0.0005%
NN-HYA03	Hyaluronidase	A0A2D4JS30	*Micrurus lemniscatus lemniscatus*	27–91	0.0003%
NN-HYA04 *	Hyaluronidase	A0A194APD1	*Micrurus tener*	1–440 (98.43)	0.0003%
**Nerve growth factor (NGF)**					**2.88%**
NN-NGF01 *	Venom nerve growth factor 2	Q5YF89	*Naja sputatrix*	1–241 (100)	2.88%
**Vespryn (VES)**					**0.04%**
NN-VES01 *	Ohanin	P83234	*Ophiophagus hannah*	1–181 (100)	0.03%
NN-VES02 *	Ohanin	P83234	*Ophiophagus hannah*	1–181 (100)	0.02%
**Vascular endothelial growth factor (VEGF)**					**0.001%**
NN-VEGF01 *	Vascular endothelial growth factor 1	U3F558	*Micrurus fulvius*	1–142 (95.95)	0.001%
NN-VEGF02 *	Vascular endothelial growth factor 1	U3F558	*Micrurus fulvius*	1–142 (95.95)	0.0003%
**Dipeptidyl peptidase IV (DPP IV)**					**0.02%**
NN-DPP01	Dipeptidyl peptidase 4	V8P9G9	*Ophiophagus hannah*	0.02%	0.02%
**Phospholipase B (PLB)**					**0.01%**
NN-PLB01 *	Phospholipase-B 81	F8J2D3	*Drysdalia coronoides*	1–553 (100)	0.01%
NN-PLB02	Phospholipase B-like	V8NLQ9	*Ophiophagus hannah*	146–196	0.001%
**Snake C-type lectin (SNACLEC)**					**0.17%**
NN-SNAC01 *	C-type lectin BfL-1	Q90WI8	*Bungarus fasciatus*	1–158 (100)	0.06%
NN-SNAC02 *	C-type lectin BfL-1	Q90WI8	*Bungarus fasciatus*	1–148 (98.67)	0.11%
NN-SNAC03	Venom C-type lectin mannose binding isoform 1 variant 3	D2YVK0	*Tropidechis carinatus*	57–158	0.001%
**Cystatin (CYS)**					**0.01%**
NN-CYS01	Cystatin	E3P6P4	*Naja kaouthia*	0.01%	0.01%
**Aminopeptidase (AP)**					**0.004%**
NN-AP01 *	Aminopeptidase	U3FZS8	*Micrurus fulvis*	1–992 (98.29)	0.003%
NN-AP02 *	Aminopeptidase	U3FZS8	*Micrurus fulvius*	1–992 (98.29)	0.001%
**Waprin (WA)**					**0.001%**
NN-WAP01	Waprin-Phi3	A7X4M7	*Philodryas olfersii*	33–80	0.001%
**Natriuretic peptide (NP)**					**0.55%**
NN-NP01	Natriuretic peptide Na-NP	D9IX97	*Naja atra*	34–77	0.41%
NN-NP02	Natriuretic peptide Na-NP	D9IX97	*Naja atra*	115–165	0.08%
NN-NP03	Natriuretic peptide Na-NP	D9IX97	*Naja atra*	25–80	0.06%
**L-amino acid oxidase (LAAO)**					**0.08%**
NN-LAAO01	L-amino-acid oxidase	Q4JHE3	*Oxyuranus scutellatus scutellatus*	1–517	0.04%
NN-LAAO02	L-amino-acid oxidase	Q4JHE3	*Oxyuranus scutellatus scutellatus*	1–517	0.02%
NN-LAAO03	L-amino-acid oxidase	Q4JHE3	*Oxyuranus scutellatus scutellatus*	1–517	0.02%

* Indicates full-length transcripts of NN-SL with >90% coverage to the annotated sequence. ^A^ indicates the length of coverage of toxin sequence when compared to the annotated sequence, and the value in bracket refers to the % coverage of full-length transcript of NN-SL. ^B^ Transcript abundance (in %) was based on FPKM (fragments per kilobase of transcript per million mapped reads) of toxin genes in NN-SL.

**Table 3:** Overview of NN-SL venom proteins sorted according to families, subtypes and their relative abundance. The identification of tryptic digested peptides was performed using MALDI TOF/TOF and nano-ESI-LCMS/MS analyses.

Protein Name	Accession Number *	Species *	Relative Abundance
**3FTX**			**72.19%**
**SNTX**			**0.86%**
Short neurotoxin 1	P01427	*Naja oxiana*	0.86%
**LNTX**			**9.61%**
Alpha-elapitoxin-Nk2a	P01391	*Naja kaouthia*	2.22%
Long neurotoxin 1	P25668	*Naja naja*	0.54%
Long neurotoxin 1	P25671	*Naja kaouthia*	6.60%
Long neurotoxin 4	P25672	*Naja naja*	0.25%
**MTLP**			**2.34%**
Muscarinic toxin-like protein 3	P82464	*Naja kaouthia*	2.16%
Muscarinic toxin-like protein 2	P82463	*Naja kaouthia*	0.18%
**WTX**			**2.46%**
Weak neurotoxin 7	P29181	*Naja naja*	0.15%
Weak neurotoxin 6	P29180	*Naja naja*	2.31%
**CTX**			**56.92%**
Cytotoxin 3	P01446	*Naja kaouthia*	0.06%
Cytotoxin 1d/1e	Q98958	*Naja atra*	7.83%
Cytotoxin-like basic protein	P62377	*Naja naja*	4.34%
Cytotoxin 4	P60303	*Naja kaouthia*	0.38%
Cytotoxin 2c	O93472	*Naja sputatrix*	37.34%
Cytotoxin 1	P01447	*Naja naja*	6.97%
**PLA_2_**			**14.58%**
Neutral phospholipase A_2_ muscarinic inhibitor	Q92084	*Naja sputatrix*	1.88%
Acidic phospholipase A_2_ 2	P15445	*Naja naja*	11.63%
Basic phospholipase A_2_ homolog 1	P10117	*Laticauda colubrina*	0.54%
Acidic phospholipase A_2_ 1	P00596	*Naja kaouthia*	0.32%
Acidic phospholipase A_2_ 2	P00597	*Naja kaouthia*	0.21%
**KSPI**			**0.10%**
Protease inhibitor 2	Unigene36921_ESM	*Hydrophis schistosus*	0.10%
**NGF**			**0.71%**
Venom nerve growth factor	P01140	*Naja naja*	0.58%
Venom nerve growth factor	P61898	*Naja atra*	0.12%
**VES**			**0.28%**
Thaicobrin	P82885	*Naja kaouthia*	0.28%
**SVMP**			**4.06%**
Zinc metalloproteinase-disintegrin-like atrase-A	D5LMJ3	*Naja atra*	3.91%
Hemorrhagic metalloproteinase kaouthiagin	P82942	*Naja kaouthia*	0.15%
**CRISP**			**3.08%**
Cysteine-rich venom protein natrin-1	Q7T1K6	*Naja atra*	3.08%
**SVSP**			**0.95%**
Snake venom serine protease NaSP	A8QL53	*Naja atra*	0.95%
**CVF**			**1.66%**
Cobra venom factor precursor	Q91132	*Naja kaouthia*	1.66%
**PDE**			**0.07%**
Phosphodiesterase	A0A194ARD7	*Micrurus tener*	0.04%
Phosphodiesterase 1	Unigene5869_Naja-sumatrana	*Naja sumatrana*	0.03%
**LAAO**			**0.55%**
L-amino acid oxidase	A8QL58	*Naja atra*	0.44%
L-amino-acid oxidase precursor	Q4JHE3	*Oxyuranus scutellatus scutellatus*	0.11%
**5’-NUC**			**0.04%**
Snake venom 5’-nucleotidase	CL3600.Contig1_NS2A	*Naja sumatrana*	0.04%
**GPX**			**0.13%**
Glutathione peroxidase	V8P395	*Ophiophagus hannah*	0.13%
**ND**			**1.61%**

* accession number and species annotated based on sequence similarity by BLAST. Abbreviations: 3FTX, three-finger toxins; SNTX, short neurotoxin; LNTX, long neurotoxin; WTX, weak neurotoxin; MTLP, muscarinic toxin; CTX, cytotoxin/cardiotoxin; PLA_2_, phospholipase A_2_; SVMP, snake venom metalloproteinase; CRISP, cysteine-rich secretory protein; CVF, cobra venom factor; SVSP, snake venom serine protease; NGF, nerve growth factor; LAAO, L-amino acid oxidase; VES, vespryn; GPX, glutathione peroxidase; KSPI, Kunitz-type serine protease inhibitor; PDE, phosphodiesterase; 5‘-NUC, 5’-nucleotidase; ND, not determined/unidentified.

**Table 4:** Lethality and antivenom neutralization of *N. naja* venoms from different geographical locales.

Snake Locality	*i.v.* LD_50_ (mg/kg)	Antivenom Used	Challenge Dose	ED_50_ (µL) ^a^	ER_50_ (mg/mL) ^b^	Neutralization ^c^ Potency (mg/mL)	Reference
Sri Lanka	0.75 (0.48–1.18)	VINS polyvalent antivenom	2.5 LD_50_	35.00	1.23 (1.11–1.37)	0.74	Current study
Sri Lanka	1.13 (0.80–1.49)	VINS polyvalent antivenom	3.0 LD_50_	96.86	0.70 (0.60–0.90)	0.47	[22]
		Instituto Clodomiro Picado polyvalent antivenom	3.0 LD_50_	195	0.40 (0.30–0.50)	0.23	
Sri Lanka	0.67 (0.48–0.99)	VINS polyvalent antivenom	5.0 LD_50_	#		-	[12]
		Bharat polyvalent antivenom	5.0 LD_50_	#		-	
Maharashtra, West India	0.73 (0.50–0.88)	Premium Serums antivenom	5.0 LD_50_	81.27	0.90 (0.74–1.08)	0.72	[94]
Punjab, North India	0.33 (0.28–0.38)	Premium Serums antivenom	5.0 LD_50_	67.37	0.49	0.39	[6]
Rajasthan, West India	2.53	Premium Serums antivenom	5.0 LD_50_	NE	NE	NE	
West Bengal, East India	0.27 (0.22–0.32)	Premium Serums antivenom	5.0 LD_50_	60.45	0.45	0.36	
Andhra Pradesh, Southeast India	0.55 (0.45–0.65)	Premium Serums antivenom	5.0 LD_50_	54.24	1.01	0.81	
Madhya Pradesh, Central India	0.22 (0.18–0.25)	Premium Serums antivenom	5.0 LD_50_	60.45	0.36	0.29	
Pakistan	0.22 (0.12–0.40)	VINS polyvalent antivenom	5.0 LD_50_	32.77	0.77 (0.69–0.85)	0.61	[8,54]

Abbreviation: *i.v.*, intravenous; LD_50_, median lethal dose; ED_50_, median effective dose; ER_50_, median effective ratio. ^a^ the dose of antivenom (μL) to neutralize the challenge dose of venom (_Χ_LD_50_) at which 50% of mice survived; ^b^ ratio of the amount of venom (mg) to the volume of the antivenom (ml) at which 50% of mice survived; ^c^ the amount of venom (mg) completely neutralized by per unit volume of antivenom (ml). # Median effective dose (ED_50_) defined differently as the amount of antivenom (μg) per g mice weight (μg/g) to neutralize the challenge dose of venom (_Χ_LD_50_), where VINS = 0.77 μg/g (95% C.I.: 0.48–1.05) and Bharat = 1.38 μg/g (0.99–1.63); 95% confidence intervals are indicated in parentheses, unreported data are denoted in dashed line, antivenom not effective toward the challenge dose of venom at a maximum volume of 166 μL is indicated as NE.

## Data Availability

Data is contained within the article or supplementary material.

## Supplementary Materials

The following are available online at https://www.mdpi.com/article/10.3390/toxins13080558/s1, Figure S1: Correlation between the venom gland transcriptome and venom proteome of NN-SL, Supplementary File S1: Venom gland transcriptomic of *N. naja* from Sri Lanka, Supplementary File S2: Mass spectrometry analysis of in-gel tryptic digested peptides of RP-HPLC fractions of Sri Lankan *N. naja* venom, Supplementary File S3: Comparative expression profile of toxin families derived from the venom gland transcriptome and venom proteome of *N. naja* from different geographical locales.
