# Supplementary material for: Elucidating the Venom Diversity in Sri Lankan Spectacled Cobra (Naja naja) through De Novo Venom Gland Transcriptomics, Venom Proteomics and Toxicity Neutralization
Source: Toxins (Basel). 2021 Aug 10;13(8):558. doi: 10.3390/toxins13080558 (PMC8402536; doi:10.3390/toxins13080558)
Supplement: Supplementary file 1 [file toxins-13-00558-s001.zip › toxins-1301217 supplementary fig.pdf]

Supplementary Elucidating the Venom Diversity in Sri Lankan Spectacled Cobra (*Naja naja*) through De Novo Venom Gland Transcriptomics, Venom Proteomics and Toxicity Neutralization

Kin Ying Wong, Kae Yi Tan, Nget Hong Tan, Gnanathasan Christeine Ariaranee and Choo Hock Tan

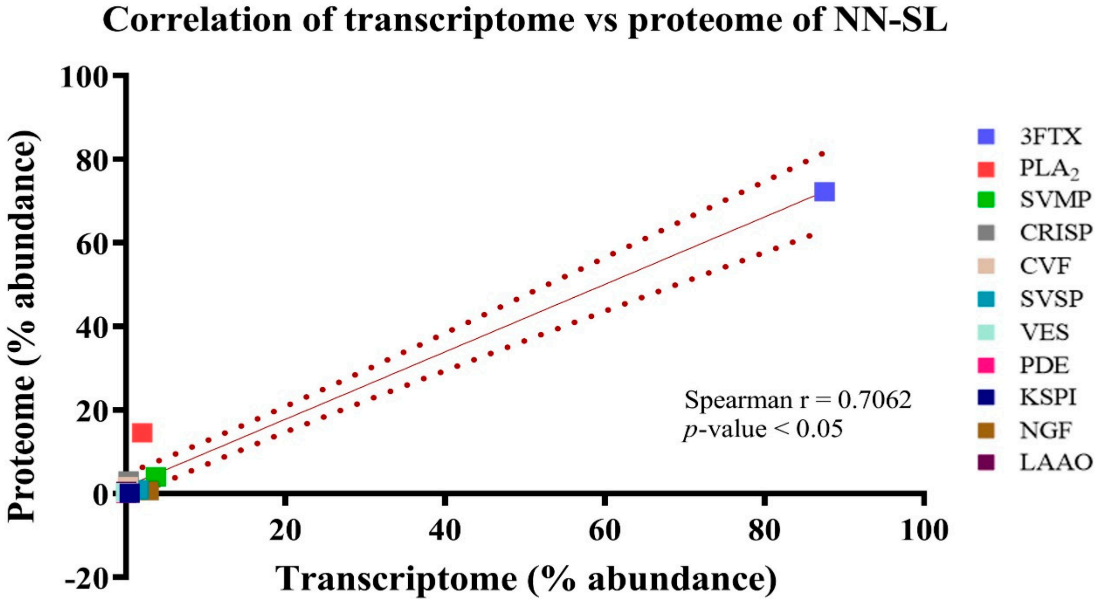

Figure S1. Correlation of proteome and transcriptome of NN-SL.
